# Single-Cell Protein and Transcriptional Characterization of Epiretinal Membranes From Patients With Proliferative Vitreoretinopathy

**DOI:** 10.1167/iovs.63.5.17

**Published:** 2022-05-17

**Authors:** Yannik Laich, Julian Wolf, Rozina Ida Hajdu, Anja Schlecht, Felicitas Bucher, Laurenz Pauleikhoff, Martin Busch, Gottfried Martin, Henrik Faatz, Saskia Killmer, Bertram Bengsch, Andreas Stahl, Albrecht Lommatzsch, Günther Schlunck, Hansjürgen Agostini, Stefaniya Boneva, Clemens Lange

**Affiliations:** 1 Eye Center, Medical Center, Faculty of Medicine, University of Freiburg, Freiburg, Germany; 2 Department of Ophthalmology, Semmelweis University, Budapest, Hungary; 3 Department of Ophthalmology, University Medical Center Greifswald, Greifswald, Germany; 4 Achim Wessing Institute for Imaging in Ophthalmology, University Hospital Essen, Essen, Germany; 5 Department of Medicine II (Gastroenterology, Hepatology, Endocrinology, and Infectious Diseases), Freiburg University Medical Center, Faculty of Medicine, University of Freiburg, Freiburg, Germany; 6 Signaling Research Centres BIOSS and CIBSS, University of Freiburg, Freiburg, Germany; 7 Institute of Anatomy and Cell Biology, Julius Maximilian University Wuerzburg, Wuerzburg, Germany; 8 Ophtha-Lab, Department of Ophthalmology at St. Franziskus Hospital, Muenster, Germany

**Keywords:** RNA sequencing, xCell analysis, imaging mass cytometry (IMC), single-cell analysis, proliferative vitreoretinopathy (PVR), epiretinal membrane (ERM), scar formation, drug repurposing

## Abstract

**Purpose:**

Proliferative vitreoretinopathy (PVR) remains an unresolved clinical challenge and can lead to frequent revision surgery and blindness vision loss. The aim of this study was to characterize the microenvironment of epiretinal PVR tissue, in order to shed more light on the complex pathophysiology and to unravel new treatment options.

**Methods:**

A total of 44 tissue samples were analyzed in this study, including 19 epiretinal PVRs, 13 epiretinal membranes (ERMs) from patients with macular pucker, as well as 12 internal limiting membranes (ILMs). The cellular and molecular microenvironment was assessed by cell type deconvolution analysis (xCell), RNA sequencing data and single-cell imaging mass cytometry. Candidate drugs for PVR treatment were identified in silico via a transcriptome-based drug-repurposing approach.

**Results:**

RNA sequencing of tissue samples demonstrated distinct transcriptional profiles of PVR, ERM, and ILM samples. Differential gene expression analysis revealed 3194 upregulated genes in PVR compared with ILM, including *FN1* and *SPARC*, which contribute to biological processes, such as extracellular matrix (ECM) organization. The xCell and IMC analyses showed that PVR membranes were composed of macrophages, retinal pigment epithelium, and α-SMA-positive myofibroblasts, the latter predominantly characterized by the co-expression of immune cell signature markers. Finally, 13 drugs were identified as potential therapeutics for PVR, including aminocaproic acid and various topoisomerase-2A inhibitors.

**Conclusions:**

Epiretinal PVR membranes exhibit a unique and complex transcriptional and cellular profile dominated by immune cells and myofibroblasts, as well as a variety of ECM components. Our findings provide new insights into the pathophysiology of PVR and suggest potential targeted therapeutic options.

Proliferative vitreoretinopathy (PVR) is a common complication of long-standing retinal detachment (RD), ocular trauma, or after surgical procedure to treat rhegmatogenous RD and can lead to blindness if left untreated.^1^ The incidence of PVR after surgical repair of RD is estimated to be 5% to 10%^2^ and has not improved notably despite great advances in vitreoretinal techniques over the past 25 years.^3^ One of the main reasons for this dilemma is the lack of an effective pharmacotherapy supporting the sophisticated vitreoretinal surgical approaches. In the past, several mostly anti-inflammatory or antiproliferative agents, including daunomycin and corticosteroids, have shown little or no effect in treating or preventing PVR in clinical trials, although showing favorable effects in experimental animal models.^3^^–^^5^

The unmet need for an adjunctive treatment is explained by our yet incomplete understanding of the molecular and cellular mechanisms underlying PVR formation in humans, which is an essential prerequisite for the development of effective pharmacotherapies. The current hypothesis for the pathogenesis of PVR assumes a multifactorial process: The initial breakdown of the retinal pigment epithelium (RPE) and the blood-retinal barrier is thought to be followed by cell migration and transdifferentiation of RPE cells into myofibroblasts and an influx of other glial and inflammatory cells. These transdifferentiating and infiltrating cells release cytokines, growth factors, and extracellular matrix (ECM) components, thus contributing to the formation of epi- and/or subretinal fibrotic membranes that can cause tractional retinal detachment and lead to irreversible blindness if left untreated.^3^^,^^6^

Previous studies investigating the molecular and cellular components associated with PVR have mainly focused on selected, predetermined molecules or signaling pathways utilizing microarray or immunohistochemical studies.^7^^,^^8^ Although these studies have provided important insights into the pathophysiology of PVR, they were limited by the techniques available at the time, which do not fully capture the complexity of the disease.

To address this issue, the current study uses state-of-the-art techniques, including RNA sequencing, single-cell protein analysis, and in silico drug repurposing to shed more light on the complex cellular and molecular nature of human PVR and to identify potential treatment options. We show that PVR membranes are characterized by a distinct transcriptional profile, in which ECM and cell adhesion components are overexpressed. In addition, PVR membranes exhibit an accumulation of various stromal and immune cells that can transdifferentiate into myofibroblasts, representing potential treatment targets to prevent disease progression.

## Materials and Methods

### Patients’ Characteristics

**Figure 1.**
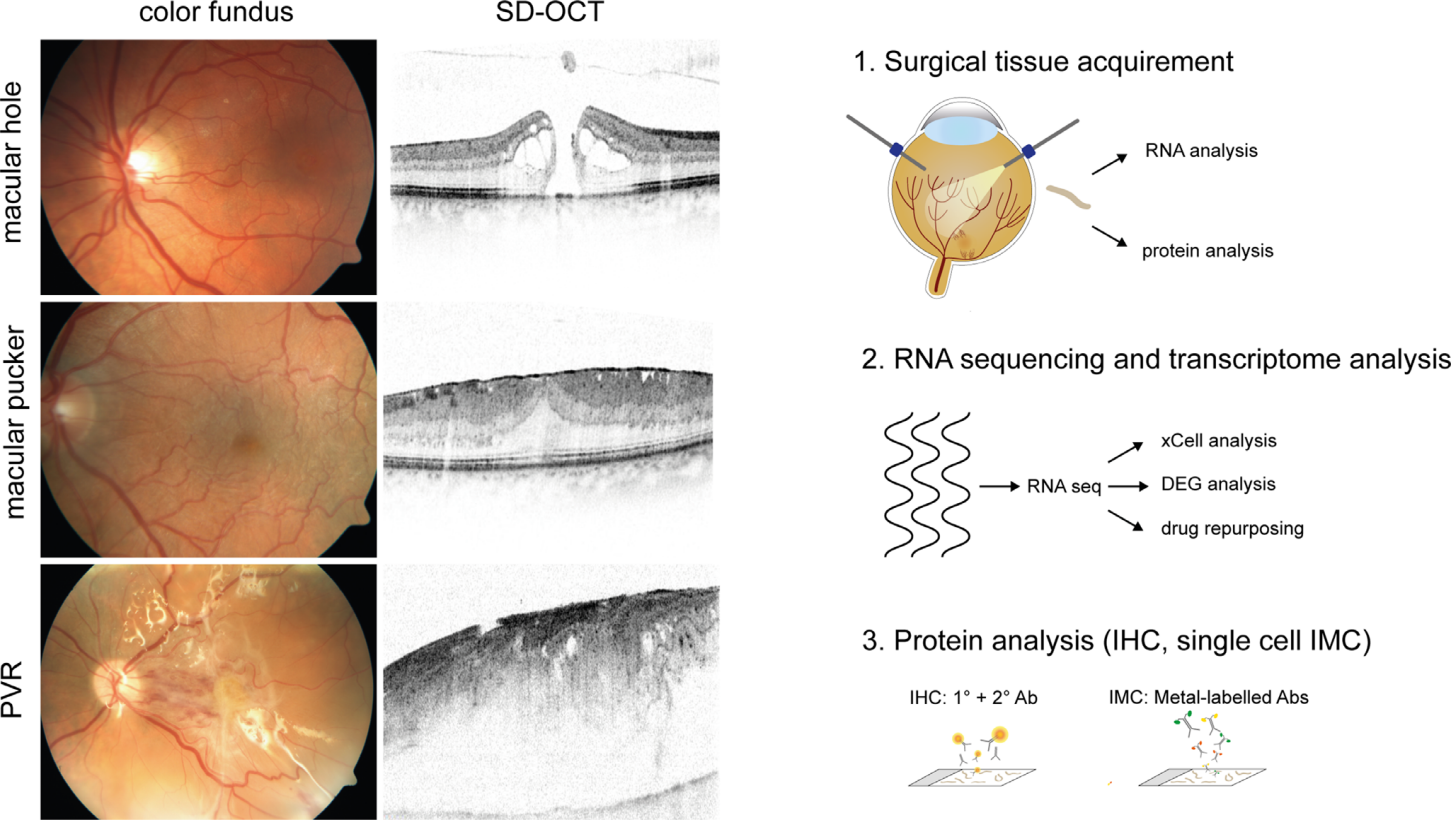
Study subjects and experimental setup. In-depth molecular characterization of internal limiting membranes (ILM), epiretinal membranes from patients with macular pucker (MP), and epiretinal membranes from patients with proliferative vitreoretinopathy (PVR). Diagnosis was made based on a thorough funduscopic examination and spectral domain optical coherence tomography (SD-OCT) imaging. Following surgical extraction, the samples were immediately processed for RNA or protein analysis. DEG, differentially expressed genes; IHC, immunohistochemistry; IMC, imaging mass cytometry; Ab, antibody.

Forty-four patients undergoing vitrectomy for idiopathic macular pucker (MP; *n* = 13), idiopathic macular hole (MH; *n* = 12), or RD due to PVR (*n* = 19) between 2019 and 2021 were included in this study (see Fig. 1, Table 1). In patients with MP, an epiretinal membrane (ERM) was removed along with the internal limiting membrane (ILM), whereas only the ILM was peeled off in patients with MH. In patients with PVR, any epiretinal proliferative vitreoretinal membranes were removed. Diagnosis was made prior to surgery based on a detailed funduscopic examination and, in case of patients with MP and MH, additional spectral domain optical coherence tomography (HRA2, Heidelberg Engineering; see Fig. 1). The study was conducted in accordance with the Helsinki Declaration. Ethics approval was granted by the local Ethics Committee and a written informed consent was obtained from all patients.

In the PVR group, there were three patients with disorders of the immune system. One patient was under infliximab and low-dose prednisolone for the treatment of systemic sarcoidosis without any signs of ocular involvement at the time of the surgery. Furthermore, a patient with Down syndrome and a patient with a mitochondriopathy due to a *mt-atp8* mutation were included in the PVR group. One of the patients with MP suffered from multiple sclerosis whereas no patients with MH had a history for an autoimmune disease. Neither of the included patients had a history of uveitis. There were several patients with diabetes mellitus type 2 (3 in the PVR group and 1 in the ILM group) but none showed signs of diabetic retinopathy, one patient in the PVR group suffered from branch retinal vein occlusion about 2 years prior to the surgery.

**Table 1.** Patients’ Characteristics

Group	ILM	ERM	*P* Value	PVR	*P* Value
*n*	12	13		19	
Age at surgery	71.1 (61.7-80.7)	70.9 (62.6-83.3)	0.99	59.6 (15.5-82.4)	0.28
Sex			0.07		0.06
Male	4 (33.3%)	9 (69.2%)		13 (68.4%)	
Female	8 (66.7%)	4 (30.8%)		6 (31.6%)	
Lens					
Phakic	7 (58.3%)	9 (69.2%)	0.57	6 (31.6%)	0.14
Pseudophakic/aphakic	5 (41.7 %)	4 (30.8%)		13 (68.4%)	
Primary PPV	11 (91.7%)	13 (100.0%)	0.29	7 (36.8%)	0.003
Hx of trauma	–	–		2 (10.5%)	
Hx of chronic RD	–	–		5 (26.3%)	
Revision PPV	1 (8.3%)	0 (0.0%)	0.29	12 (63.2%)	0.003
Without oil	1 (8.3%)	–		2 (10.5%)	
With oil	–	–		10 (52.6%)	
Staining agent	12 (100.0%)	13 (100.0%)	1.00	10 (52.6%)	0.005
PVR grading					
C1	–	–		1 (5.3%)	
C2	–	–		13 (68.4%)	
C3	–	–		5 (26.3%)	

Age at surgery is shown as mean and minimum/maximum. The percentage of patients undergoing primary pars plana vitrectomy (PPV) with a history (Hx) of trauma or long-standing chronic retinal detachment (RD) is reported. PVR patients undergoing revision PPV for retinal detachment despite initial surgical repair are subdivided into eyes that were filled with or without silicone oil prior to the procedure. The percentage of processes in which a staining agent (MembraneBlue-Dual, D.O.R.C. International, Zuidland, The Netherlands or Brillant Peel, Fluoron GmbH, Ulm, Germany) was used is indicated. Grading of proliferative vitreoretinopathy (PVR) was performed according to the updated Retina Society Classification (1991) (1). The *P* value was calculated using Mann-Whitney test for age and Chi-square test for sex, lens, blue dye, and primary and revision PPV.

PPV, pars plana vitrectomy; Hx, history; RD, retinal detachment; PVR, proliferative vitreoretinopathy.

Further details about the included specimens and demographic data of all patients are summarized in Table 1.

### RNA Extraction, Library Preparation, and RNA Sequencing

For RNA sequencing, 10 epiretinal PVR membranes from 10 patients, 10 ERM from 10 patients with MP, and 7 ILM samples from 7 patients with MH were studied. RNA extraction, library preparation, and RNA sequencing were performed at the Genomics Core Facility “KFB - Center of Excellence for Fluorescent Bioanalytics” (University of Regensburg, Germany; www.kfb-regensburg.de) as previously described.^9^^–^^11^ In short, tissue samples for RNA analysis were directly transferred into 50 µl RNA*later* (Thermo Fisher). RNA*later* was subsequently replaced by RLT Plus buffer and the obtained tissue was homogenized by vortexing for 30 seconds. Genomic DNA contamination was eliminated using gDNA Eliminator spin columns. After ethanol addition, the samples were applied to RNeasy MinElute spin columns followed by several wash steps. Last, total RNA was eluted in 12 µl of nuclease-free water, and 750 pg of total RNA was converted to first-stranded cDNA using the SMARTer Ultra Low Input RNA Kit for Sequencing version 4 (Clontech Laboratories, Inc.). To amplify double-stranded cDNA 12 cycles of LD-PCR were performed, followed by purification via magnetic bead clean-up. Library preparation was carried out according to Illumina Nextera XT Sample Preparation Guide (Illumina, Inc.). One hundred fifty (150) pg of cDNA were tagged and fragmented via Nextera XT transposome. After adding partial adapters, a limited-cycle PCR program was used for amplification resulting in multiplexed sequencing libraries. The libraries were quantified with the KAPA SYBR FAST ABI Prism Library Quantification Kit (Kapa Biosystems, Inc.). The Illumina TruSe SR Cluster Kit version 3 was used for cluster generation on the cBot with pooled equimolar amounts of each library. The products were sequenced on a HiSeq1000 instrument with TruSeq SBS Kit version 3 according to the Illumina HiSeq 1000 System User Guide. Illumina image analysis and base calling were recorded and converted to Fastq files via the CASAVA1.8.2/ bcl2fastq version 2.18 software. The sequencing data are available in the Gene Expression Omnibus Database under the following accession number: GSE179603.

### Bioinformatics

Sequencing data (fastq files) were uploaded to and analyzed on the Galaxy web platform (usegalaxy.eu),^12^ as previously described.^13^^,^^14^ Quality control was performed via FastQC Galaxy version 0.72 (http://www.bioinformatics.babraham.ac.uk/ last accessed on October 8, 2020). Reads were mapped to the human reference genome (Gencode, release 35, hg38, all) by RNA STAR Galaxy Version 2.7.5b^15^ with default parameters using the Gencode main annotation file (Gencode, release 38, https://www.gencodegenes.org/human/releases.html). Reads mapped to the human reference genome were counted via feature Counts Galaxy version 1.6.4^16^ with default parameters using the aforementioned annotation file. The output of featureCounts was imported to RStudio (version 1.4.1103 and R version 4.0.3). Gene symbols and gene types were determined based on ENSEMBL release 101 (human genes, download on October 28, 2020).^17^ Genes with zero reads in all samples were removed from analysis. Principal component analysis (PCA)^18^ was applied to check for potential batch effects. Differential gene expression was analyzed using the R package DESeq2 version 1.30.1^18^ with default parameters (Benjamini-Hochberg adjusted *P* values, lfcShrink: type = “normal”). Transcripts with log2 fold change >2 or <−2 and adjusted *P* value <0.05 were considered as differentially expressed genes (DEGs). Heatmaps were created with the R package ComplexHeatmap 2.6.2.^19^ Other data visualization was performed using the ggplot2 (3.3.3) package.^20^ Based on the significantly upregulated genes in PVR tissue, Gene Ontology enrichment analysis and its visualization were carried out using the R package clusterProfiler 3.18.1^21^ with default parameters. Genes associated with the five most significantly enriched biological processes in PVR tissue were illustrated using the R function cnetplot of the clusterProfiler package. Cell type enrichment analysis was performed using xCell.^22^ The tool utilizes the transcriptomic signatures of 64 distinct immune and stroma cell types to estimate their relative contributions to a bulk RNA transcriptome. Transcripts per million were calculated as an input for the analysis based on the output of featureCounts (assigned reads and feature length), as previously described.^23^ The xCell enrichment scores were compared between different groups using the Mann–Whitney *U* test.

### Transcriptome-Based Drug-Repurposing

In quest of so far unidentified therapeutic options for PVR, we applied a transcriptome-based drug-repurposing approach, similar to previously described strategies.^24^^,^^25^ In a first step, significantly upregulated genes in PVR when compared to ILM were determined, as described above. To identify the most relevant PVR-associated factors, we next analyzed known interactions between PVR genes using STRING analysis^26^ and retained for further analysis only genes with at least one known interaction. Based on the identified potential therapeutic targets, a drug search was performed in the drug database drugbank.ca^27^ with the following filter criteria: target organism = “humans,” target known action = “yes,” and group = “approved.” In a next step, we used drug-exposure transcriptome data from the CMAP database^28^ as a reference and screened each candidate drug-induced transcriptional profile in relation to our PVR signature. For this purpose, all genes expressed in PVR membranes were ranked according to their log2 fold change compared to the ILM expression profile. If the genes downregulated by the drug ranked at the top of the PVR gene expression list, the given drug-induced profile was complementary to the expression profile related to the disease and might therefore be a potential treatment option for PVR. The accuracy of fit was quantified by calculating an enrichment score and an adjusted *P* value using Gene Set Enrichment Analyses (GSEA).^29^

### Immunohistochemistry

For immunohistochemical analysis, 5 additional epiretinal PVR membranes and 5 ILM from 10 patients were stabilized in 4% paraformaldehyde (PFA) on ice for 1 hour and, after extensive rinsing with PBS to remove any potential residuals of the staining agent (MembraneBlue-Dual, D.O.R.C. International, Zuidland, Netherlands or Brillant Peel, Fluoron GmbH, Ulm, Germany) used during the surgery, transferred into 20% sucrose diluted in 0.027 M PBS. Following embedding in optimal cutting compound medium (Tissue-Tek O.C.T, Sakura) and freezing, 10-µm sections were prepared in a cryostat and stored at -20°C until staining. Sections were blocked with 1% bovine serum albumin (BSA; Roth) and 5% normal donkey serum (NDS; Jackson Immuno Research) in 0.027 M PBS with 0.3% Triton X-100 (Sigma-Aldrich) for 60 minutes at room temperature. Primary antibodies against Tyrosinase-related protein 1 (TYRP1; 1:200; Abcam; ab235447), alpha smooth muscle actin (α-SMA; 1:500; Sigma-Aldrich; A2547) ionized calcium-binding adaptor molecule 1 (IBA-1; 1:500; Abcam, ab5076), cluster of differentiation (CD) 206 (1:5000; Abcam, ab64693), Fibronectin (FN1; 1:200; Sigma-Aldrich, F6140), and secreted protein acidic and rich in cysteine (SPARC; 1:200; Sigma-Aldrich; HPA002989) were diluted in the blocking solution. Sections were incubated with the primary antibody solution overnight at 4°C. Primary antibodies were omitted in negative controls. Following extensive rinsing with PBS, sections were stained with an Alexa Fluor 568-coupled donkey anti-goat, an Alexa Fluor 488-coupled donkey anti-mouse, or a Cyanine Cy 5-conjugated donkey anti-rabbit secondary antibody (diluted 1:500 in the blocking buffer) at room temperature for 1 hour. After washing at least 3 times with PBS, sections were counterstained with 4′,6-Diamidin-2-phenylindol (DAPI; 1:1000; Sigma-Aldrich) for 10 minutes, washed 3 times with PBS, followed by autofluorescence quenching with TrueBlack Lipofuscin Autofluorescence Quencher (Biotium) according to the manufacturer's protocol. Images were taken on a Leica TCS SP8 Confocal System coupled to a Leica DMi8 inverted microscope equipped with 20 times (0.75 NA) and 40 times (0.95 NA) air objectives.

### Imaging Mass Cytometry

Imaging mass cytometry (IMC) was performed on PVR and ERM specimens as previously described.^30^ Because IMC was not feasible on ILM samples due to the small tissue size and low cell numbers, 3 ERM samples were adduced for the comparison to 4 epiretinal PVR membranes. In brief, tissue samples were fixed in 4% formalin for 12 hours after excision and dehydrated by ascending ethanol series (70%, 80%, 2 × 95% for 30 minutes and 2 × 100% for 15 minutes). After 2 incubation steps in xylene (1 hour each), the samples were incubated in liquid paraffin for 4 hours and subsequently embedded. For staining, 6 µm thick sections were prepared.

Prior to staining, paraffin slides were heated at 60°C for 90 minutes and deparaffinized in xylene 2 times for 10 minutes followed by rehydration in a descending ethanol series (2 × 100%, 95%, and 80% for 5 minutes each). After washing with tris-buffered saline (TBS) for 5 minutes, the slides were incubated in a pressure cooker with DAKO EnvisionFlex target retrieval solution (high pH; Agilent Technologies) at 95°C for 30 minutes to perform heat-induced antigen retrieval. After cooling down and washing with TBS, slides were blocked in 3% BSA in TBS for 60 minutes at room temperature. A customized panel of antibodies (Fluidigm) was used to stain the sections. A list of antibodies, clones, and conjugated metals is shown in Table 2. 1:100 (VEGF, Arginase 1) or 1:800 (other antibodies) diluted antibodies were applied to sections simultaneously within an antibody mix and incubated overnight at 4°C in a hydration chamber. After incubation over night at 4°C in a hydration chamber, the slides were washed with TBS 3 times for each, 5 minutes each, and treated with iridium-intercalator solution (1:2000 in TBS) for 5 minutes followed by 3 washing steps in TBS for 5 minutes each. After 30 minutes of drying at room temperature, preparation for laser ablation and image acquisition was completed.

**Table 2.** Targets, Clones, and Conjugates Used for Imaging Mass Cytometry

Target	Clone	Metal
αSMA	1A4	141Pr
EGFR	D38B1	142Nd
Vimentin	D21H3	143Nd
CD16	EPR16784	146Nd
CD163	EDHu-1	147Sm
PanKer	C11	148Nd
CD11b	EPR1344	149Sm
CD274	SP142	150Nd
CD31	EPR3094	151Eu
CD45	D9M8I	152Sm
CD44	IM7	153Eu
B-Actin	2F1-1	154Sm
E-Cadherin	24E10	158Gd
CD68	KP1	159Tb
CD8a	C8/144B	162Dy
VEGF	G153-694	163Dy
Arginase	D4E3M	164Dy
CD74	LN2	166Er
GranzB	EPR20129-217	167Er
Ki67	B56	168Er
Collagen I	Polyclonal	169Tm
Histon3	D1H2	171Yb
CD276	Polyclonal	173Yb
HLA DR	LN3	174Yb
Pan-Actin	D18C11	175Lu

### Image Acquisition

Image acquisition was performed with the Hyperion Imaging System (Fluidigm) according to the manufacturer's instructions. After determination of the regions of interest by dark-field microscopy, the tissue sections were laser-ablated spot-by-spot at 200 Hz resulting in a pixel size/resolution of 1 µm². Multiple 1500 µm × 1500 µm images per sample were acquired. Raw data was processed using the CyTOF software version 7.0 (Fluidigm). MCD Viewer version 1.0.560.6 (Fluidigm) was used to view the images.

### Segmentation and High-Dimensional Data Analysis

The staining pattern of each antibody was checked for feasibility in all samples. The following antibodies showed plausible results and were included in further analyses: α-SMA, vimentin, CD16, CD163, CD45, CD44, β-actin, CD68, CD8a, vascular endothelial growth factor (VEGF), arginase I, CD74, Ki-67, collagen I, histone H3, CD276, and Human Leukocyte Antigen DR (HLA-DR). IMC data were analyzed as described previously.^30^^,^^31^ In short, the acquired mcd files were converted into tiff image stacks using a Python script adapted from https://github.com/BodenmillerGroup/ImcSegmentationPipeline. Subsequently, segmentation masks were set using the *ilastik* software^32^ (version 1.3.2) to identify nuclei, cytoplasm, and background fractions prior to uploading the probability maps into *CellProfiler*^33^ (version 3.1.8). The generated cell masks were used to extract single-cell information and subsequently uploaded into *histoCat*^34^ (version 1.76) to calculate mean marker pixel intensity. The data was normalized to the 99th percentile for PhenoGraph clustering^35^ (nearest neighbors = 15). Clustering was performed based on data from the above-mentioned markers showing a plausible staining. Further analysis of the single-cell cluster data was conducted using Omiq.ai (Omiq). To visualize the cellular profile on a single-cell basis *opt*-SNE (optimized t-Distributed Stochastic Neighbor Embedding) dimensionality reduction^36^ was performed with the following settings: arcsinh transformation cofactor: 0.2, max iterations: 1000, *opt*-SNE end: 5000, perplexity: 30, theta: 0.5, random seed: 1535, and verbosity: 25. Omiq was used to analyze marker expression in specific Phenograph clusters, followed by visualization as a heatmap using ComplexHeatmap 1.20.0^19^ in RStudio (version 1.4.1103, R version 4.0.3). Cluster assembly between entities was plotted as bargraphs using the *ggplot2* package.^20^ Subsequent statistical analysis was performed in GraphPad Prism (GraphPad Software, version 6.0).

## Results

### Transcriptional Characterization of PVR Membranes

**Figure 2.**
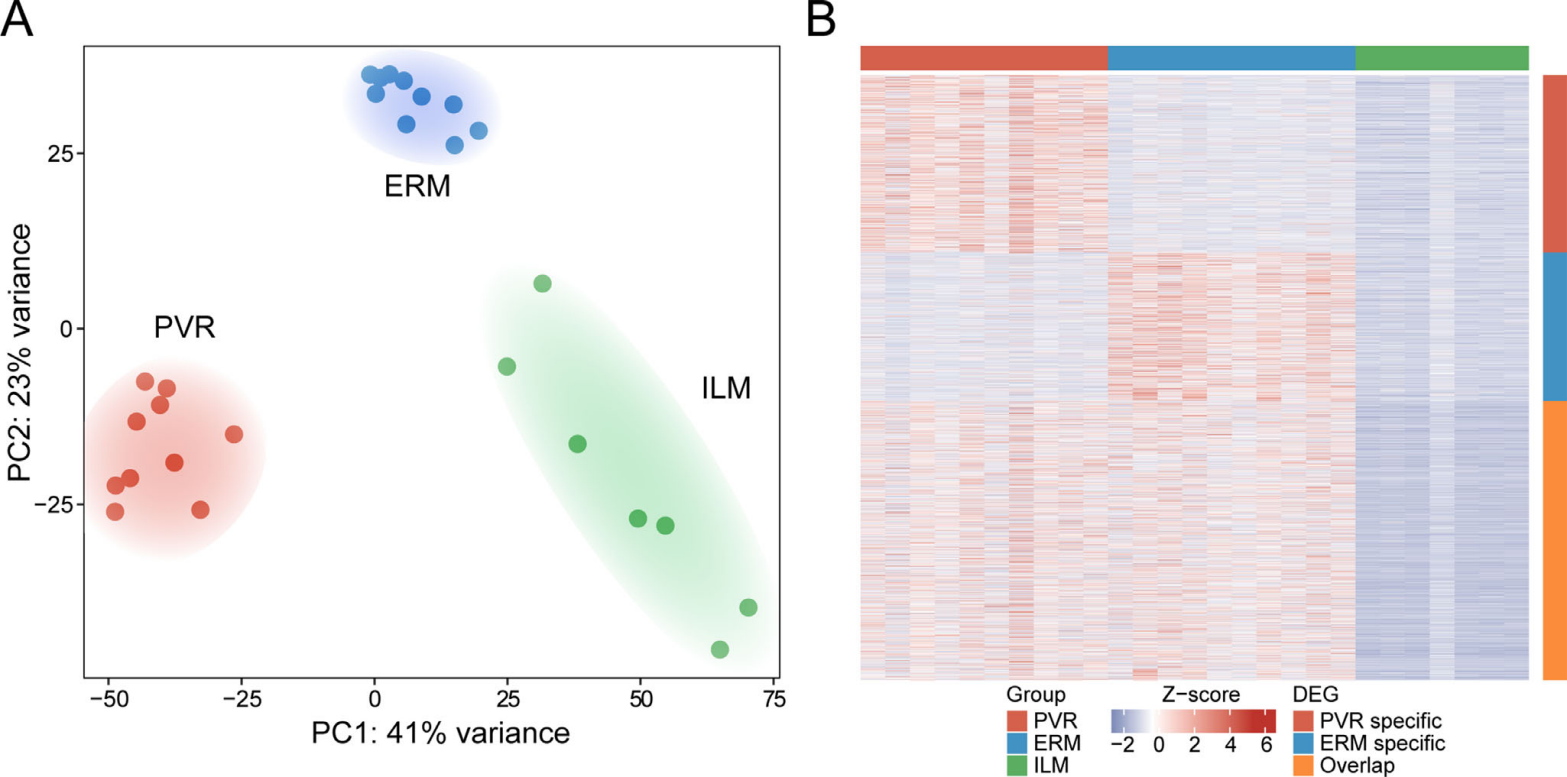
Transcriptional characterization of PVR, ERM, and ILM tissue. (**A**) The unsupervised clustering of the transcriptional profiles of PVR, ERM, and ILM samples in a principal component analysis (PCA) reveals an accurate distinction of the three tissue types. (**B**) Heatmap visualizing tissue-specific genes in PVR and ERM samples, each compared to ILM samples. Each column represents one sample and each row represents one gene (refer to colored legend for different tissue types). The z-score represents a gene's deviation in relation to its mean expression in all samples in standard deviation units (*red* = upregulation and *blue* = downregulation).

**Figure 3.**
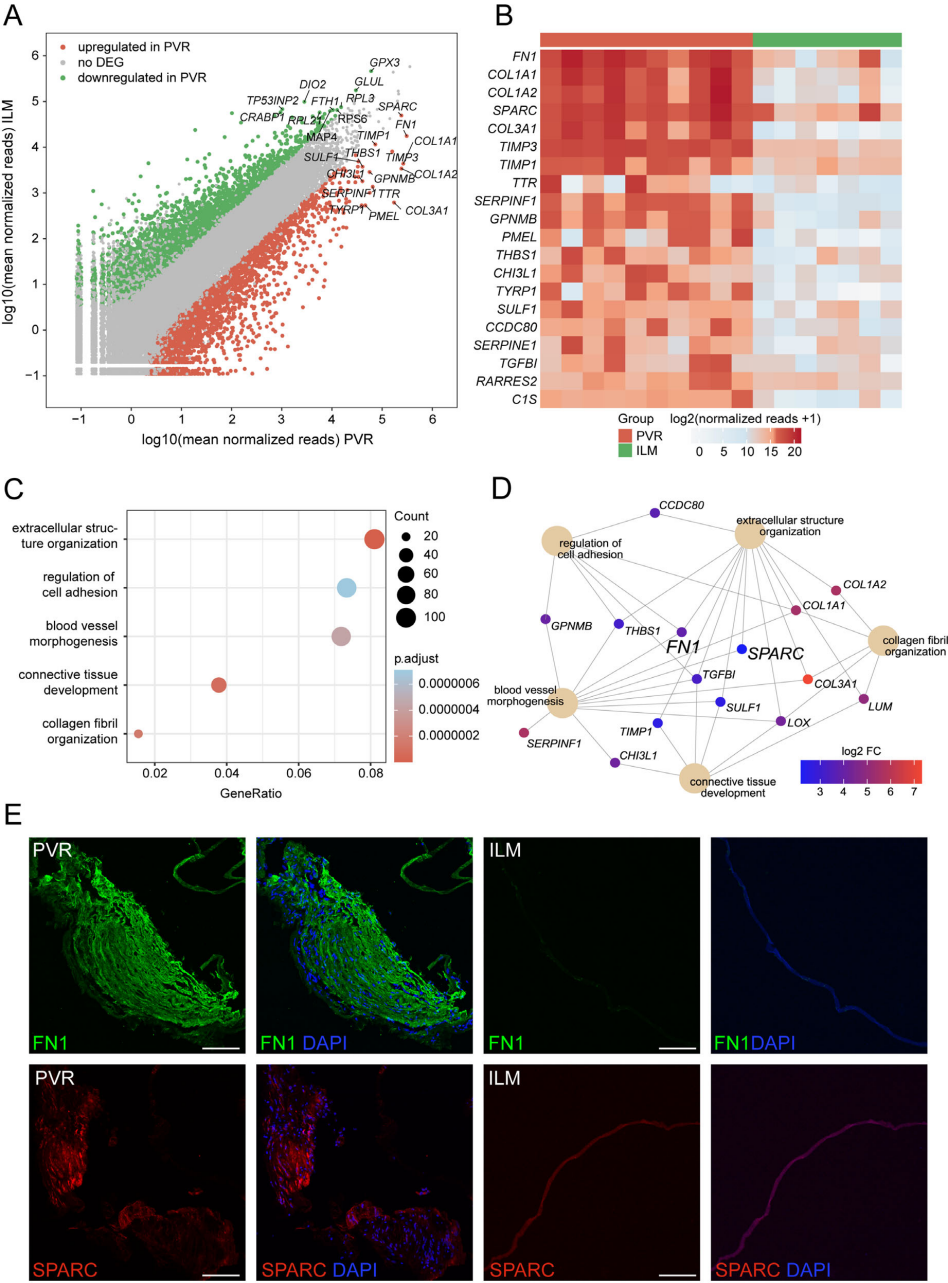
Differentially expressed genes in PVR membranes. (**A**) Readplot showing the up- and downregulated DEG (*green* and *red dots*, respectively) and similarly expressed genes (*grey dots*) according to the log2 fold change and the adjusted *P* value in PVR when compared to ILM. The top expressed DEG according to the mean expression in each group are labeled. (**B**) Supervised heatmap depicting the top PVR-specific genes when compared to ILM. (**C**) GO enrichment analysis based on the 3194 significantly enriched genes in PVR membranes. Dot plot illustrating the top five enriched biological processes ordered by the number of DEG associated with the GO term (count). The size of the dots represents the count, and the dots’ colors represent the adjusted *P* values. The gene ratio describes the ratio of the count to the number of all DEG. (**D**) The genes associated with the 5 most significantly disease-associated GO biological processes are illustrated in the cnetplot, with the color representing each DEG's log2 fold change. (**E**) Immunofluorescence staining for FN1 (*upper panel*) and SPARC (*lower panel*) in PVR and ILM samples. Nuclei are counterstained with 4',6-Diamidino-2-phenylindol (DAPI). Scale bars correspond to 100 µm.

RNA sequencing revealed pronounced transcriptional differences between PVR, ERM, and ILM tissue samples, as apparent from the unsupervised clustering of the three different entities in the principal component analysis (see Fig. 2A) and the high number of DEGs between the groups visible in the heatmap (see Fig. 2B). DEG analysis revealed 3194 up- and 2639 downregulated genes in PVR when compared to ILM (see Fig. 3A) and 856 up- and 901 downregulated genes in PVR when compared to ERM (see Supplementary Fig. S1A). Among the former, *FN1* (Fibronectin 1), *COL1A1* (alpha-1 type I Collagen), *COL1A2* (alpha-2 type I Collagen), *SPARC* (Osteonectin), and *COL3A1* (alpha-1 type III Collagen) were the five highest expressed DEG in PVR (see Fig. 3B). Gene ontology (GO) analysis demonstrated that these DEGs contributed to biological processes, such as extracellular structure organization (GO: 0043062), regulation of cell adhesion (GO: 0030155), blood vessel morphogenesis (GO: 0048514), connective tissue development (GO: 0061448), and collagen metabolic process (GO: 0032963; see Fig. 3C). According to the network diagram in Figure 3D illustrating the linkages of DEG and GO terms, transforming growth factor beta induced (*TGFBI*), *FN1*, *SPARC*, and different types of collagens emerged as central factors in these biological processes (see Fig. 3D). In addition, factors including *LOX* (Lysyl oxidase), *CHI3L1* (Chitinase 3 Like 1), and *LUM* (Lumican), as well as *THBS1* (Thrombospondin 1), *GPNMB* (Glycoprotein Nmb), and *CCDC80* (Coiled-Coil Domain Containing 80), were found to contribute to connective tissue development and cell adhesion regulation, respectively. Similarly, the top DEG between PVR and ERM tissue included *COL1A1*, *COL1A2*, *COL3A1*, *TIMP3* (metallopeptidase inhibitor 3), and *EFEMP1* (epithelial growth factor-containing fibulin-like extracellular matrix protein 1), mostly contributing to biological processes, such as chemotaxis (GO: 0006935), angiogenesis (GO: 0001525), leukocyte migration (GO: 0050900), regulation of hormone levels (GO: 0010817), and collagen metabolic process (GO: 0032963; see Supplementary Fig. S1B, 1C). Consistent with the increased number of *FN1* and *SPARC* transcripts in human PVR membranes, we found a strong immunofluorescent signal for FN1 and SPARC in PVR membranes compared to ILM, which affirmed the sequencing results on the protein level (see Fig. 3E, Supplementary Fig. S2).

### Cellular Composition of PVR, ERM, and ILM Samples

**Figure 4.**
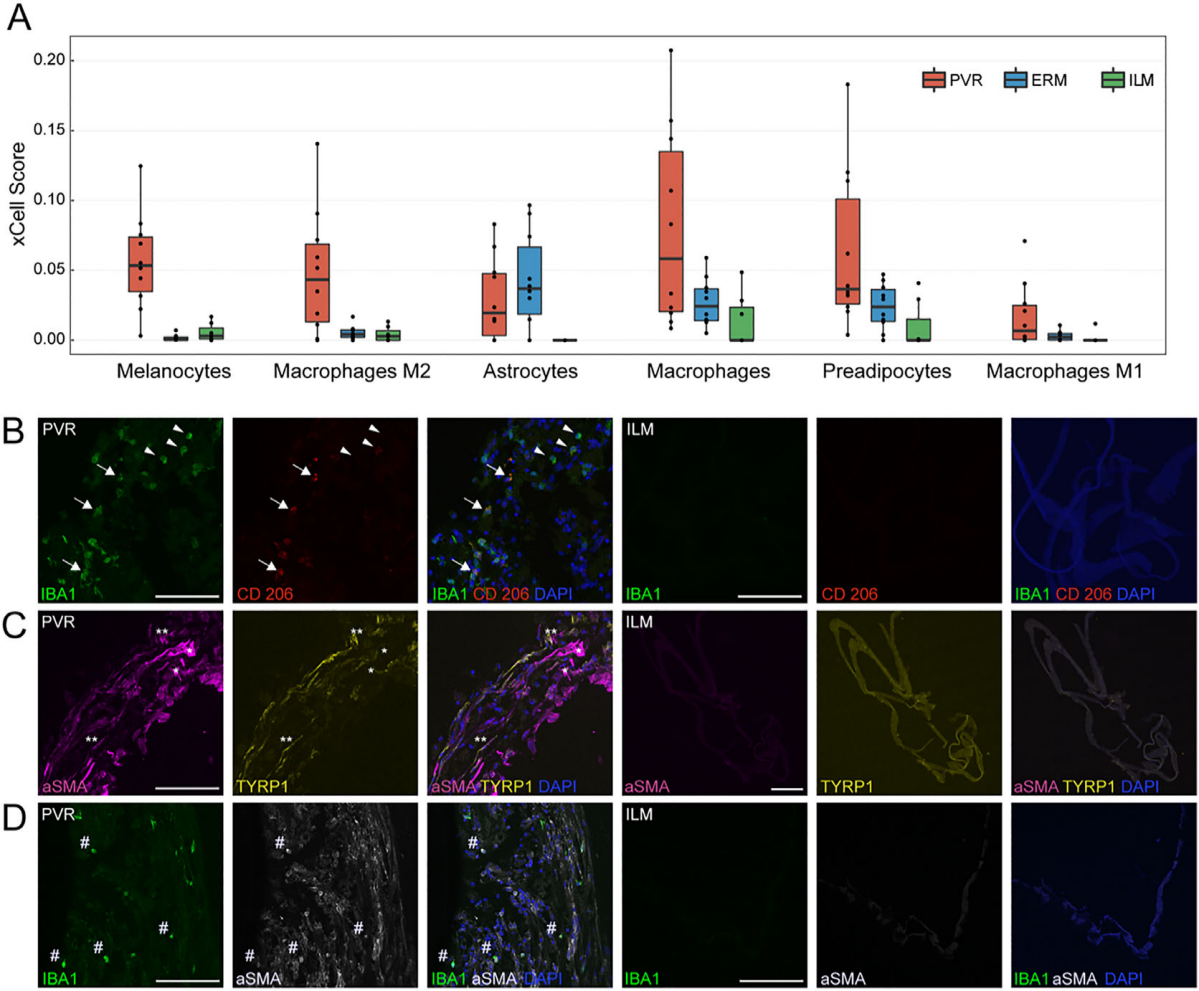
Cellular composition of PVR, ERM, and ILM samples. (**A**) Cell type enrichment analysis of the sequencing data via xCell: six cell types were significantly enriched in PVR when compared to ILM tissue **A**. (**B, C,** **D**) Immunofluorescence staining of PVR (*n* = 5) and ILM (*n* = 5) specimens confirming the presence of IBA1-positive macrophages and CD206-positive M2 macrophages in PVR membranes, which are absent in ILM control specimens. Some of the IBA1-positive macrophages (*arrows*) co-expressed CD206 (*arrow heads*) suggesting a M2 polarization **B**. TYRP1-expression (melanocytes/RPE cells) was observed in one out of five PVR membranes and colocalized with α-SMA (*double asterisks*) pointing toward an epithelial-to-mesenchymal transdifferentiation of RPE cells to myofibroblasts. A subset of the α-SMA-positive cells, however, were negative for TYRP1 (*single asterisk*) pointing toward alternative cellular origins of myofibroblasts in PVR **C**. All five PVR membranes revealed IBA1-positive myeloid cells, such as hyalocytes, microglia, or macrophages, which co-expressed α-SMA (hashtag) suggesting a transdifferentiation of myeloid cells to myofibroblasts as a common pathophysiological feature during PVR formation **D**. Nuclei are counterstained with DAPI. Scale bars correspond to 100 µm.

To explore the cellular components that might contribute to the transcriptional changes in PVR membranes described above, we next performed cell type enrichment analysis using xCell.^22^ This analysis revealed several cell types enriched in PVR membranes when compared to ERM and ILM samples. Among them, melanocytic cells, M2 macrophages, and astrocytes were most significantly increased in PVR compared to ILM (xCell scores: melanocytic cells: PVR: 0.05 [0.03 – 0.07], ILM: 0.003 [0.001 – 0.009], *P* < 0.003; M2 macrophages: PVR: 0.04 [0.01 – 0.06], ILM: 0.003 [0.000 – 0.007], *P* < 0.02; astrocytes: PVR: 0.02 [0.003 – 0.04], ILM: 0.000 [0.000 – 0.000], *P* < 0.009, median [interquartile range], Mann–Whitney *U* test; see Fig. 4A). To validate these findings on the protein level, we next assessed the expression of TYRP1 (a marker for melanocytic cells and RPE cells), IBA1 (a marker for myeloid cells), and CD206 (a marker for M2 macrophages), as well as α-SMA (a marker of myofibroblast formation) by immunofluorescence staining. In line with the sequencing results, we found a strong immunoreactivity for IBA1 and CD206 in PVR membranes when compared to ILM suggesting a role of IBA1/CD206-positive macrophages in the pathogenesis of PVR (see Fig. 4B, Supplementary Fig. S3). TYRP1-expression was observed in one out of five stained PVR membranes and colocalized with α-SMA pointing toward a possible epithelial-to-mesenchymal transdifferentiation of RPE cells to myofibroblasts in this patient (see Fig. 4C, Supplementary Fig. S3). However, numerous α-SMA-positive myofibroblasts were negative for TYRP1, raising the question about alternative cellular origins of myofibroblasts abundant during PVR formation (see Fig. 4C, Supplementary Fig. S3). Interestingly, all five PVR membranes revealed cells that co-expressed IBA1 and α-SMA, suggesting a transdifferentiation of myeloid cells to myofibroblasts as a common pathophysiological feature during PVR formation (see Fig. 4D, Supplementary Fig. S3).

**Figure 5.**
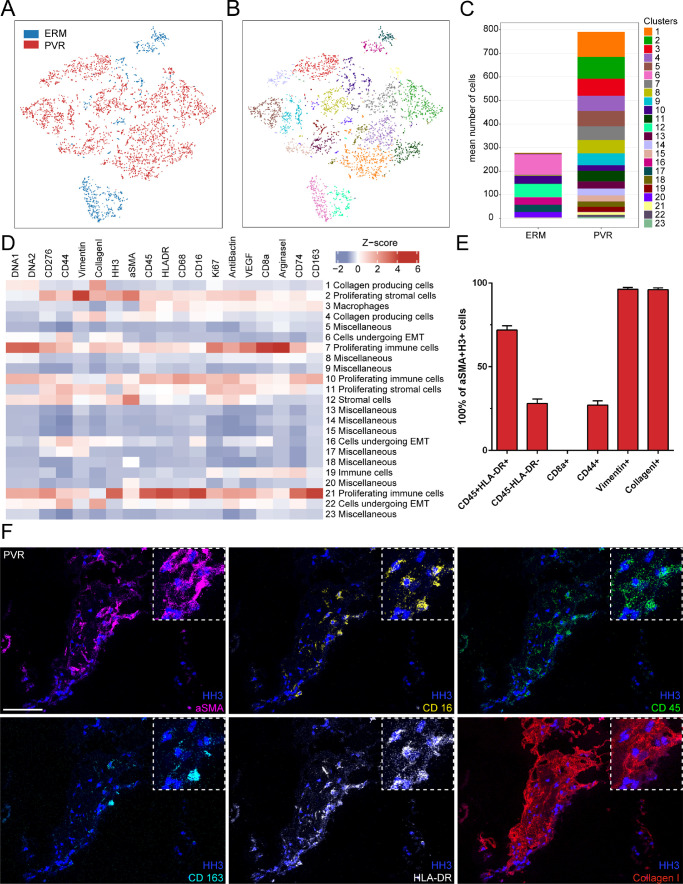
Imaging mass cytometry of PVR membranes. (**A,** **B**) Phenographic clustering of the high-dimensional single-cell IMC data revealed a total of 23 distinct cell clusters in PVR **A** (*red dots*) and ERM tissue samples **A** (*blue dots*). (**C**) Cluster assembly was compared between PVR and ERM and visualized in stacked bar charts displaying mean counts per group. (**D**) Heatmap of marker signal intensity in the Phenograph clusters depicted in **B**. Z-score: deviation from a marker's mean expression in standard deviation units. Annotation of clusters was performed according to specific marker expression. (**E**) Bar plot showing the proportion of CD45-/HLA-DR, CD8a-, CD44-, vimentin-, and collagen-1-positive cells in the α-SMA-positive cell population. (**F**) Representative multiplexed staining for α-SMA (α-smooth muscle actin, *magenta*), CD16 (*yellow*), CD45 (*green*), CD163 (*light blue*), HLA-DR (human leukocyte antigen - DR, *white*), and collagen1 (*red*) on a PVR sample. Nuclei are stained with HH3 (histone H3, *dark blue*). Scale bar corresponds to 100 µm.

To gain further and more detailed insight into the cellular components contributing to the above-described transcriptional profiles in PVR formation, we next performed IMC on PVR tissue. Because the ILM is mostly acellular and thus not suitable for IMC, we used ERM as control tissue in this series of experiments. Using this technique, we simultaneously analyzed the spatial distribution of 26 proteins in PVR and ERM tissue (see Fig. 5). A total of 18 proteins showing plausible and robust staining patterns were included in subsequent bioinformatics analysis (see Supplementary Fig. S4). Following image acquisition, supervised machine learning was exploited for image segmentation and protein quantification according to a previously published protocol.^30^^,^^31^ Phenographic clustering of the high-dimensional single-cell IMC data revealed a total of 23 distinct cell clusters in PVR and ERM tissue samples. Among them, 18 clusters could be assigned to PVR, whereas only 5 clusters (clusters 6, 12, 16, 17, and 20) could be allocated to ERM (see Figs. 5A–C). The most enriched clusters in PVR were annotated according to their expression profile as collagen-producing cells (clusters 1 and 4), proliferating stromal cells (clusters 2 and 11), macrophages (cluster 3), cells undergoing epithelial-to-mesenchymal transition (clusters 6, 16, and 22), and proliferating immune cells (clusters 7, 10, and 21; see Fig. 5D). Because some of the PVR-specific cell clusters, such as clusters 2, 4, 7, and 21, were characterized by the expression of α-SMA, a classic marker for myofibroblasts implied in scar formation,^37^ we examined the α-SMA-positive cells in more detail in quest of their origin. We found that 71.9% (±2.6%) of the α-SMA-positive cells co-expressed the immune cell markers CD45 and HLA-DR, whereas 28.1% (±2.6%) were negative for these markers. None of the α-SMA-positive cells expressed the T-cell marker CD8, whereas 27.1% (±2.6%) of α-SMA-positive cells expressed CD44 suggestive for an epithelial-to-mesenchymal transition.^38^ Interestingly, nearly all α-SMA positive cells expressed vimentin (96.3% ±1.1%) and collagen 1 (96.6% ±1.1%), highlighting the role of myofibroblasts in the production of extracellular matrix proteins in PVR (see Fig. 5E). Consistent with these results, we observed a substantial number of α-SMA-positive cells that also expressed collagen 1, as well as the immune cell markers CD16, CD45, CD163, and HLA-DR (see Fig. 5F).

### Drug Repurposing

**Figure 6.**
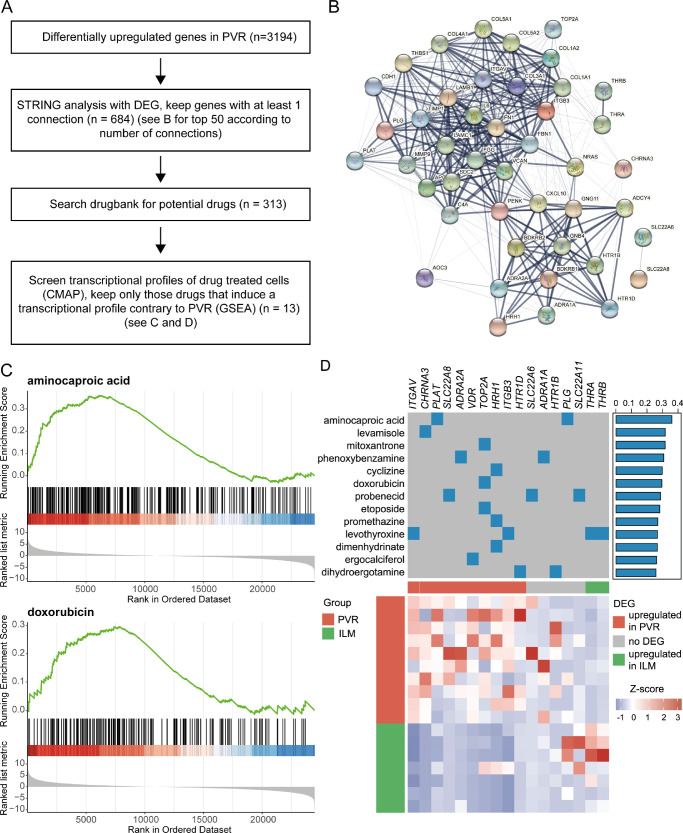
Transcriptome-based drug repurposing. (**A**) Overview of analysis. (**B**) String network of the top PVR factors with the highest number of interactions. (**C**) Enrichment score curves of the Gene Set Enrichment Analysis for two fitting drugs, namely aminocaproic acid and doxorubicin. The ordered data set at the bottom represents the log2 fold change-ranked list of PVR genes. Each gene being downregulated by the respective drug is shown by a *vertical black line* in the center row of the plot. This visualizes at which position of the PVR-ranked list the drug-regulated genes are located. The more the downregulated genes are located on the left side of the plot in the area of most upregulated PVR genes, the more contrary the drug-induced gene expression profile is in relation to the disease and, therefore, could be a potential treatment option for PVR. The accuracy of fit is quantified by the enrichment score, which is defined by the peak of the *green line*. (**D**) Heatmap of most fitting identified drugs. The upper part of the heatmap displays the drugs in the rows and their known targets in the columns, as well as the normalized enrichment score from GSEA to the right. The lower part of the heatmap visualizes the expression of the targets shown above in PVR and control tissue. Each row represents one sample and each column represents one target gene. The z-score represents a gene's expression in relation to its mean expression by standard deviation units (*red* = upregulation and *blue* = downregulation).

Finally, in search for new therapeutic options for PVR, we applied a transcriptome-based drug-repurposing approach based on our RNA-sequencing results, as previously described^24^^,^^25^ (see Fig. 6A). In brief, we identified a PVR gene signature using STRING interaction analysis^26^ (see Fig. 6B) and subsequently identified drugs with known targets within this gene signature.^27^ In order to identify the most appropriate of these agents for PVR treatment, we used drug-exposure transcriptome data^28^ in relation to the PVR profile and identified 13 drugs, which induce contrary transcriptional modulations in PVR tissue and might therefore be a potential treatment option for PVR (see Figs. 6C, 6D and refer to Methods for details). As a result, aminocaproic acid was identified as the best-fitting agent (normalized enrichment score: 1.58, adjusted *P* < 0.0001), followed by several topoisomerase 2A inhibitors, among them mitoxantrone (normalized enrichment score: 1.37, adjusted *P* < 0.01), doxorubicin (normalized enrichment score: 1.31, adjusted *P* < 0.03), and etoposide (normalized enrichment score: 1.27, adjusted *P* < 0.02).

## Discussion

PVR remains an unresolved clinical challenge in the management of retinal detachment and can lead to frequent revision surgery or even blindness due to the lack of efficient drug therapies supporting sophisticated surgical approaches. Deciphering the cellular and molecular mechanisms underpinning PVR formation is fundamental to understanding and effectively treating the disease. In the past, clinical and histological studies have shown that PVR membranes are characterized by excessive wound-healing responses and enriched in infiltrating glia and immune cells, resulting in sub-, intra-, or epiretinal scarring.^3^ Although these studies have provided important insights into the pathophysiology of PVR, they were often limited by the use of conventional immunohistochemical techniques focusing on single predetermined proteins and cells, which cannot adequately capture the complex picture of PVR. To address this issue, the current study combines RNA sequencing analysis, cell type data deconvolution, and single cell mass cytometry imaging to provide an unbiased quantitative assessment of the gene expression profile and simultaneous multiplex protein measurement of 18 cellular markers at subcellular resolution in human PVR tissue.

The comprehensive transcriptional analysis of human PVR membranes, ERMs, and ILMs revealed considerable differences in RNA expression among these entities. A total of 3194 and 856 differentially expressed transcripts were identified that were significantly increased in human PVR membranes compared with ILM and ERM control tissue, respectively. According to the conducted GO analysis, the PVR-associated factors were critical in fundamental processes in PVR pathology, such as “extracellular structure organization,” “regulation of cell adhesion,” and “connective tissue development.” These results imply an interplay between various factors expressed by diverse cell types that promote undesirable epiretinal membrane formation.^39^ As such, we detected many profibrotic and ECM-modulating factors, including *FN1* (Fibronectin 1), *TIMP1* and *3*, *SPARC*, and various collagen types, to be significantly increased in human PVR membranes, which is consistent with the literature.^40^^–^^43^ In particular, *FN1* expression ranked highest among all DEG, supporting the notion of its central role in PVR formation and unraveling potential treatment opportunities.^44^^,^^45^ Both the plasma form of FN, which circulates in the blood and is incorporated into fibrin clots upon tissue injury, as well as cellular FN have been reported to accumulate in PVR^46^^,^^47^ and to be essential for the transdifferentiation of α-SMA-positive myofibroblasts.^45^^,^^48^ Thus, the inhibition of fibronectin self-association and the fibronectin cell-binding domain, by, for example, intravitreal injection of single-chain variable fragment antibodies, may become a therapeutic option to reduce PVR as suggested by in vitro studies.^47^ Whereas the above-mentioned factors have already been linked with PVR, the current study uncovers novel cellular molecular mediators of human PVR, such as Lumican (*LUM*), Chitinase-3-like protein 1 (*CHI3L1*), or transforming growth factor beta-induced (*TGFBI*), to name a few, which have received less attention and need to be further investigated for their pathophysiological role and therapeutic potential in future studies.

In accordance with the prevailing notion, the RNA sequencing and cell deconvolution analysis of this study point toward an abundance of melanocytic cells, probably RPE cells, but also several immune cell populations in PVR membranes. Immunohistochemical analysis of human PVR membranes confirmed the presence of macrophages and in particular CD206-positive M2 macrophages, which are considered to act in an anti-inflammatory manner and promote tissue remodeling and repair.^49^ These results are consistent with preclinical data showing an accumulation of M2 macrophages in murine PVR, which were successfully modulated by intravitreal inhibition of the Notch pathway, thereby attenuating PVR formation.^50^ Although the basic dichotomous view of M1/M2 macrophages is very simplistic and should rather be considered as a continuum,^51^ M2 macrophages might represent an interesting target for immunomodulatory approaches in PVR. The high abundance of myeloid cells in PVR prompted us to study these cells in more detail using single-cell imaging mass cytometry. Phenographic clustering of the high-dimensional single-cell IMC unraveled several PVR-specific myeloid cell clusters expressing immune and antigen-presenting markers (CD45, HLA-DR, CD74, and CD276), as well as cell proliferation markers (Ki67). In addition, we found numerous α-SMA-positive myofibroblasts in human PVR membranes that invariably expressed ECM components, such as vimentin and collagen, supporting the predominant view of them as important cellular mediators of retinal fibrosis and progression of PVR.^52^^,^^53^ Interestingly, about 70% of the α-SMA-positive cells in our analysis also expressed common myeloid signature markers, such as CD45 and HLA-DR, indicating a myeloid cell to myofibroblast transdifferentiation in PVR membranes, as recently described for renal fibrosis.^54^ However, we cannot rule out with certainty that activated RPE cells that accumulate in PVR membranes adopt an immune cell-like phenotype and express markers, such as CD45 or CD68, as previously reported in vitro.^55^^,^^56^ However, the proximity of epiretinal PVR membranes to resident myeloid cells, such as retinal microglia and in particular vitreal hyalocytes,^13^^,^^57^ makes it very likely that myeloid cells contribute to the myofibroblast pool and thus modulate human PVR formation. This hypothesis is supported by clinical evidence showing that vitreous cortex remnants caused by vitreoschisis and incomplete surgical removal of the vitreous and harboring hyalocytes, are a predisposing risk factor for the development of postoperative PVR.^57^^,^^58^ Future studies are needed to determine with certainty the origin and exact role of myeloid cells in PVR membranes and to evaluate their potential as therapeutic targets.

Finally, in search of an effective pharmacological agent for the prevention or treatment of PVR, we applied a CMap-based drug repurposing strategy.^28^ Next to known antiproliferative drugs that have already been investigated in clinical trials on PVR, such as daunomycin,^4^ we identified several alternative agents that so far have not been linked to PVR treatment. Among them, aminocaproic acid, levamisole, and TOP2A inhibitors, such as etoposide, mitoxantrone, and doxorubicin, ranked among the best matching substances. Aminocaproic acid (ACA) is an antifibrinolytic medication that competitively inhibits plasminogen activation to plasmin by binding to the Kringle domain of plasminogen and subsequently leading to a reduction in fibrinolysis. In the context of PVR, it is interesting to note that plasmin is known to induce smooth muscle cell proliferation.^59^ In addition, epsilon-aminocaproic acid prevents cell growth, migration, and invasion in vitro^60^ suggesting a beneficial effect in the treatment of PVR. On the other hand, topoisomerase 2 is an enzyme essential for DNA replication, chromosome condensation, and segregation, and its inhibition is exploited in the therapy of many neoplasms, including breast, lung, and testicular cancers.^61^ The expression of *TOP2A* and *TOP2B* was significantly increased in PVR compared to ILM in our data, which indicates that topoisomerase 2-mediated cell proliferation is a fundamental pillar of PVR formation. Although the topoisomerase inhibitor daunorubicin has already been tested for the treatment of PVR in clinical trials and failed to improve the outcome,^4^ little is known about the effects of other TOP2 inhibitors, such as doxorubicin, etoposide, and mitoxantrone in PVR. Preclinical studies suggest that liposomal doxorubicin or etoposide can reduce PVR formation in an experimental rabbit PVR model without causing detectable neurotoxic side effects,^62^^,^^63^ a notion, which implies topoisomerase 2 inhibitors as effective adjunctive treatment options for the prevention of PVR. Finally, levamisole emerged as a drug of interest in our analysis for treating PVR. At high doses, levamisole has both immunosuppressive and antineoplastic features and can potentiate the anti-proliferative effect of 5-fluorouracil in several types of tumor cell lines in a dose-dependent manner.^64^ It is important to note that levamisole has cytotoxic effects by inducing apoptosis, as evidenced by increases in the levels of DNA fragmentation and the activation of caspase-3 activity in myeloma cells.^65^ Therefore, particular care must be taken when exploring the effect of levamisole to treat PVR, which ideally would be feasible by an intravitreous application, thus reducing the likelihood of systemic complications. Another agent that suppresses inflammation and inhibits cell proliferation is methotrexate, which is currently being tested in a phase III trial for the prevention of PVR (GUARD trial, NCT04136366) based on positive data from pilot clinical and in vitro studies.^66^^–^^68^ Our drug repurposing analysis identified methotrexate as a potential substance that might be beneficial for the treatment of PVR with a normalized enrichment score of 1.08 in the GSEA. However, this value was significantly lower than the score of our best fitting substances and did not reach a significant level with an adjusted *P* value of 0.13. The clinical results of the GUARD study are therefore eagerly awaited to determine whether our in silico drug repurposing approach is consistent with the clinical trial results.

In conclusion, the current study characterizes the complex cellular and molecular interactions in human PVR to an unprecedented extent by using bulk RNA sequencing, single-cell protein analysis, and in silico approaches for drug repurposing. The distinct transcriptional profile of PVR membranes is characterized by a number of immunological factors and extracellular matrix components, as well as an accumulation of various stromal, immune cells, and myofibroblasts. A subset of myofibroblasts exhibited characteristic immune cell signatures, suggesting that immune cells contribute to the myofibroblast cell pool, paving the way for potential immunomodulatory treatment approaches to prevent disease progression. This study thus provides new insights into the pathophysiology of human PVR, reveals numerous targets for the development of targeted PVR diagnostics, and lays the groundwork for future therapeutic trials.

## Supplementary Material

Supplement 1
