# Supplementary material for: Single-Cell Protein and Transcriptional Characterization of Epiretinal Membranes From Patients With Proliferative Vitreoretinopathy
Source: Invest Ophthalmol Vis Sci. 2022 May 17;63(5):17. doi: 10.1167/iovs.63.5.17 (PMC9123517; doi:10.1167/iovs.63.5.17)
Supplement: Supplement 1 [file iovs-63-5-17_s001.pdf]

SUPPLEMENTAL FIGURES

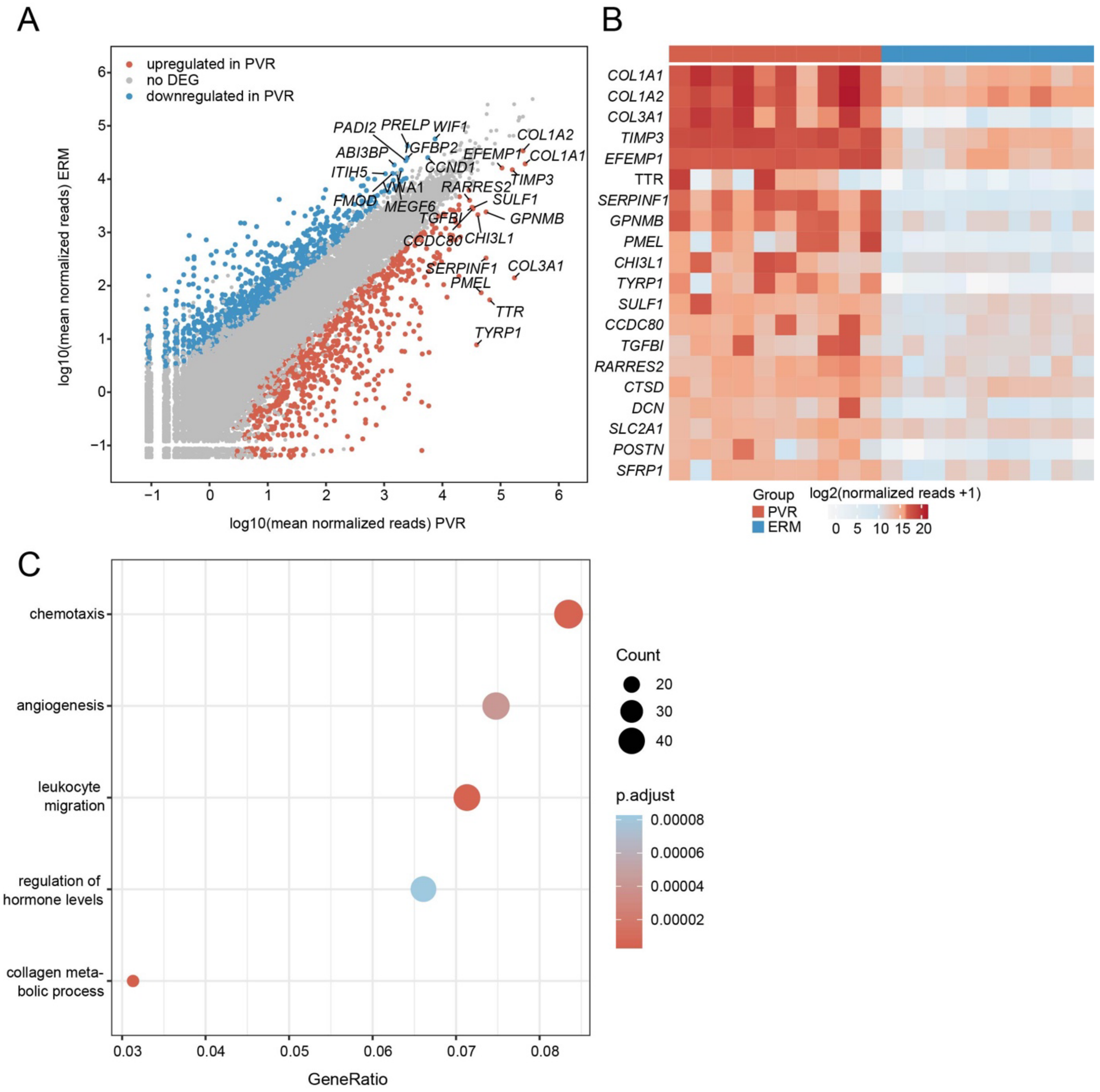

Supplemental Figure 1: Transcriptional characterization of PVR membranes in comparison to ERM. (**A**) Readplot showing the up- and downregulated DEG (red and blue dots, respectively) and similarly expressed genes (grey dots) according to the log2 fold change and the adj. p-value between PVR and ERM. The top 10 DEG according to mean expression in PVR membranes and ERM are labeled. (**B**) Supervised heatmap depicting the top PVR-specific differentially expressed genes when compared to ERM. (**C**) GO enrichment analyses for the 856 differentially expressed genes in PVR. Dot plot depicting the top five enriched biological processes ordered by the number of DEG associated with the GO term (count). The size of the dots represents the count, and the dots' colors represent the adj. p-values. The gene ratio describes the ratio of the count to the number of all DEGs.

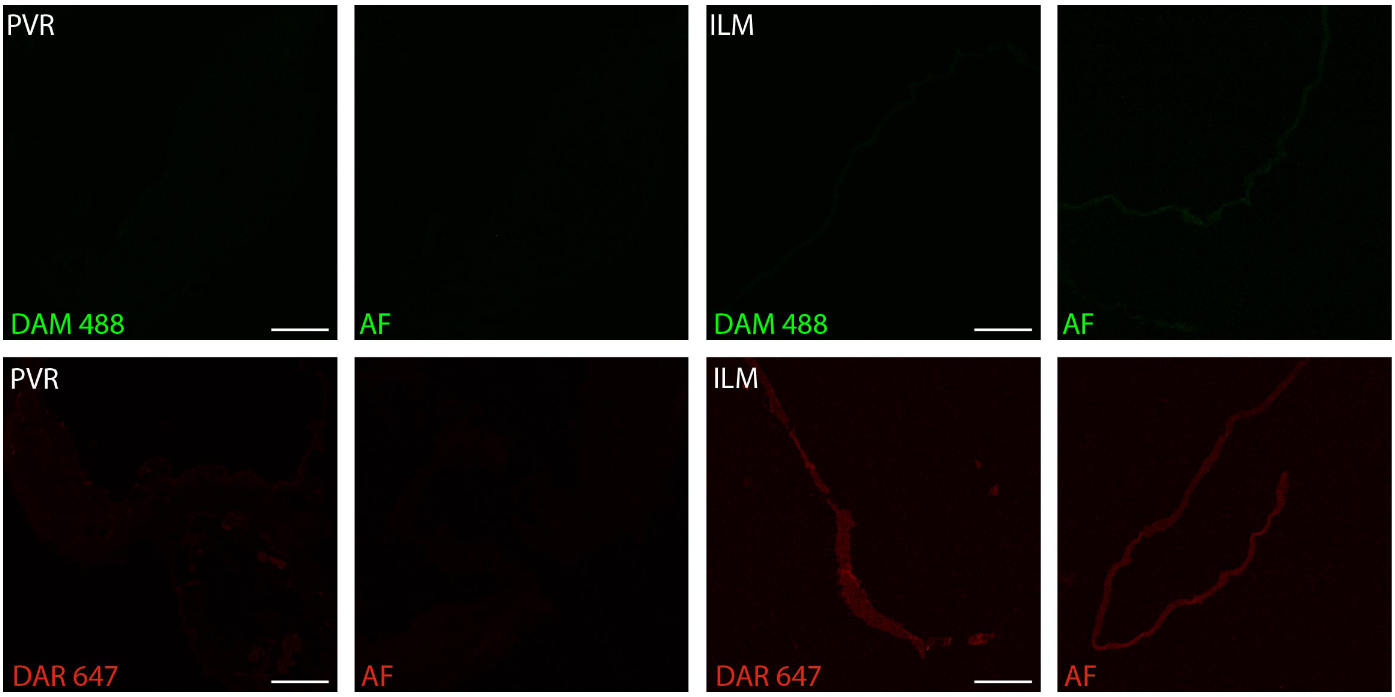

Supplemental Figure 2: Negative and autofluorescence controls for immunohistochemistry. Negative controls, omitting the primary antibodies, for FN1 (Fibroncetin 1) and SPARC (secreted protein acidic and rich in cysteine) immunohistochemical stainings (shown in Fig 3E) in proliferative vitreoretinopathy (PVR) and internal limiting membrane (ILM) tissue samples. Donkey anti-mouse 488 (DAM 488), donkey anti-rabbit 647 (DAR 647). Autofluorescence (AF) controls for IBA1, CD206, a-SMA and TYRP1 immunohistochemical stainings in PVR and ILM (shown in Fig 3E). Scale bars correspond to 100 µm.

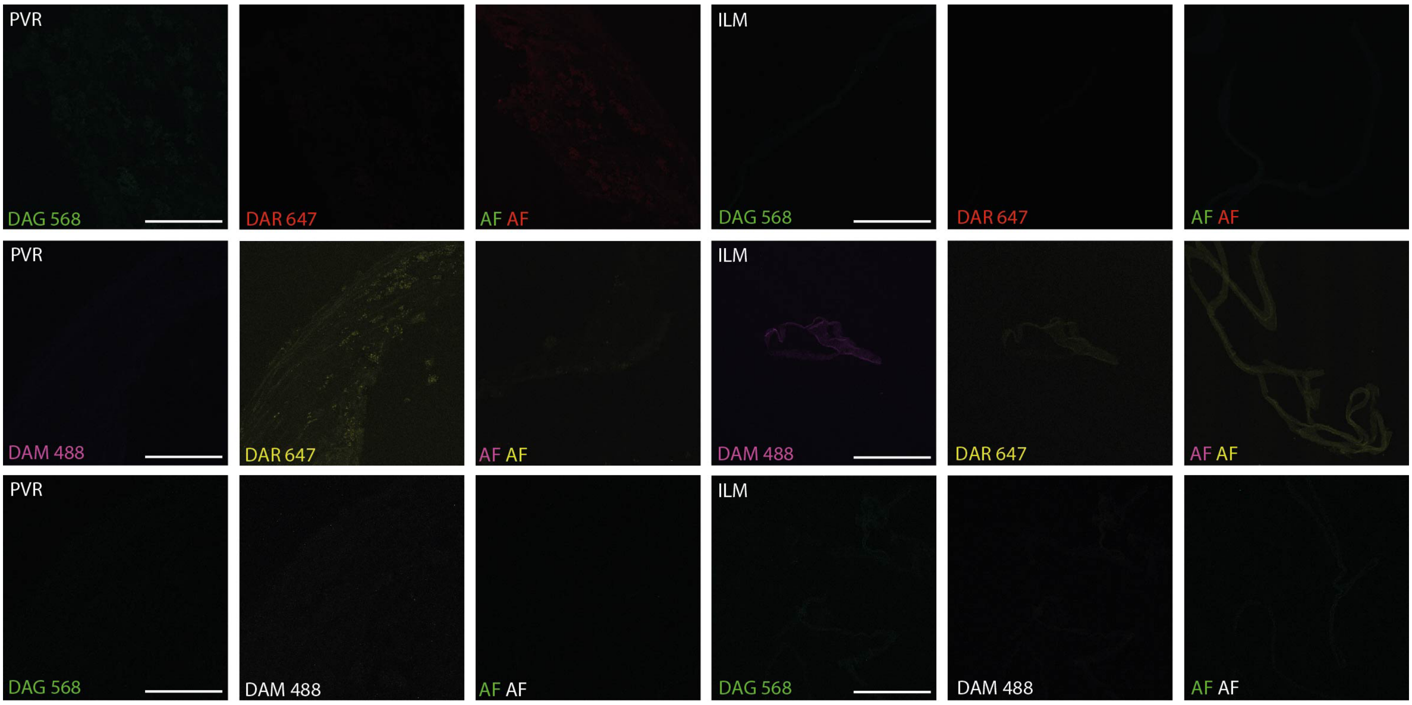

Supplemental Figure 3: Negative and autofluorescence controls for immunohistochemistry. Negative controls, omitting the primary antibodies, for IBA1 (ionized calcium-binding adapter molecule 1), CD206 (Cluster of Differentiation 206), α-SMA (α-smooth muscle actin) and TYRP1 (Tyrosinase-related protein 1) immunohistochemical stainings (shown in Fig 4B-D) in proliferative vitreoretinopathy (PVR) and internal limiting membrane (ILM) tissue samples. Donkey anti-goat 568 (DAG 568), donkey anti-rabbit 647 (DAR 647), donkey anti-mouse 488 (DAM 488). Autofluorescence (AF) controls for IBA1, CD206, a-SMA and TYRP1 immunohistochemical stainings in PVR and ILM (shown in Fig 4B-D). Scale bars correspond to 100 µm.

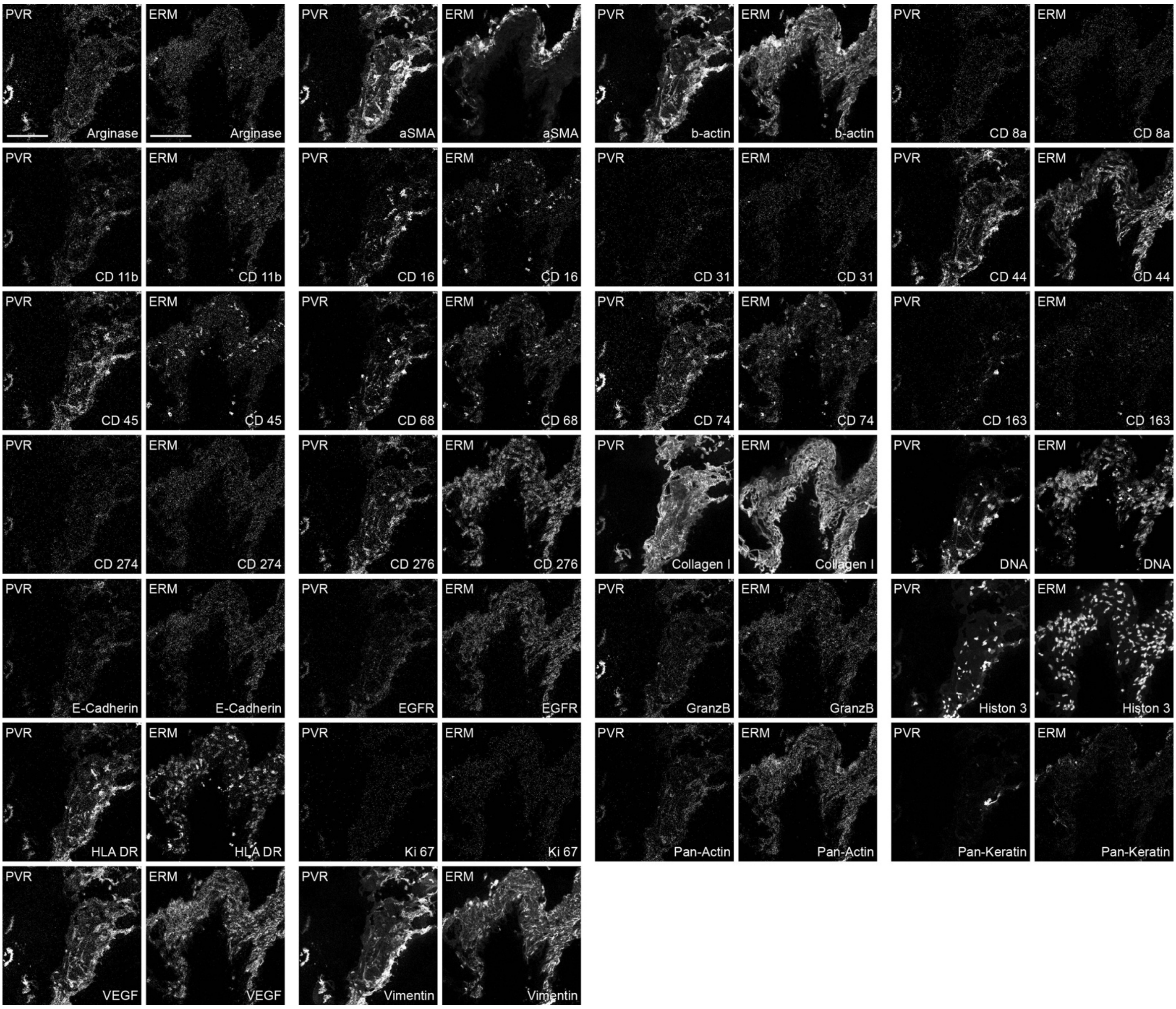

Supplemental Figure 4: Overview of Imaging Mass Cytometry. Imaging mass cytometry showing the spatial distribution of 26 markers in each a single section of proliferative vitreoretinopathy (PVR) and epiretinal membrane (ERM) samples.
